# Observational case-control study of small-fiber neuropathies, with regards on smoking and vitamin D deficiency and other possible causes

**DOI:** 10.3389/fmed.2022.1051967

**Published:** 2023-01-12

**Authors:** Maxime Fouchard, Emilie Brenaut, Steeve Genestet, Anne-Sophie Ficheux, Pascale Marcorelles, Laurent Misery

**Affiliations:** ^1^Department of Dermatology, CHU Brest, Brest, France; ^2^Univ Brest, LIEN, Brest, France; ^3^Department of Neurology, CHU Brest, Brest, France; ^4^Breton Competence Center of Rare Neuromuscular Diseases and Neuropathies With Cutaneous-Mucosal Symptoms, CHRU de Brest, Brest, France; ^5^Department of Pathology, CHU Brest, Brest, France

**Keywords:** small-fiber neuropathy, pruritus, smoking, vitamin D, pain

## Abstract

**Introduction:**

Small fiber neuropathies (SFNs) are disorders of skin nerve endings inducing pruritus, burning pain, numbness, and paresthesia. The aims of this study were to search for putative etiologies of SFN and their occurrence in a cohort of patients and to compare patients with SFN to a group of patients without SFN to highlight potential factors associated with SFN.

**Methods:**

This study was observational, retrospective, and monocentric. All patients with symptoms of SFN who underwent skin biopsies with intraepidermal nerve density counts were included. Patients with a count lower than 5 percentiles were considered to be in the SFN group. Other patients were considered to be the control group.

**Results:**

A total of 162 patients with SFN and 161 controls were included. No cause was identified for 108 patients (61.7%). The established causes were autoimmune diseases (9.1%), diabetes or glucose intolerance (8%), medication (4%), liver disease (3.4%), and monoclonal gammopathy of undetermined significance (2.9%). Current or former smokers were more numerous in the SFN group (26.5%) than in the control group (16.1%), while vitamin D amounts were significantly lower in the SFN group than in the control group.

**Discussion:**

Hence, tobacco smoking and vitamin D deficiency might be new putative causes of SFN.

## Introduction

Small-fiber neuropathies (SFNs) are disorders of unmyelinated C-fibers and poorly myelinated A-delta fibers that induce pruritus and other cutaneous paresthesia (1, 2). A recent systematic literature review showed an unmet need for broadly accepted diagnostic criteria, although the most common set of mandatory criteria to diagnose were sensory symptoms (60% of studies), pain (19% of studies), SFN signs (20% of studies), absence of large-fiber neuropathy signs (62% of studies), reduced intraepidermal nerve fiber density (IENFD) (38% of studies), and autonomic symptoms (1% of studies) (3). Nonetheless, the joint task force of the European Federation of Neurological Societies (EFNS) and the Peripheral Nerve Society (PNS) agreed to consider that distal leg skin biopsy with quantification of the IENFD, using generally agreed upon counting rules, is a reliable and efficient technique to assess the diagnosis of SFN (4). The morphometric analysis of IENFD should always refer to normative values matched for age, which were previously defined (4).

The putative causes of SFN are numerous: diabetes, autoimmune diseases (Sjögren disease, lupus, systemic sclerosis, celiac disease, and sarcoidosis), drugs, HCV or HIV infection, B6 or B12 vitamin deficiency, and hereditary causes (such as Fabry disease) (5–7). In half of the cases, SFN remain idiopathic (6). The aim of this study was to analyze the putative causes of SFN in a cohort of patients and their frequency. The second objective was to compare patients with SFN to a group of patients without SFN to identify etiological factors associated with SFN.

## Patients and methods

This study was observational, retrospective, and monocentric. Patients were selected from data of the pathology department that lists all skin biopsies performed to diagnose SFN through the evaluation of the IENFD in patients with pruritus and other cutaneous paresthesia. The period of inclusion was from June 1, 2015 to August 20, 2019.

Inclusion criteria were as follows:

• Patients who underwent a skin biopsy in the presence of symptoms that could suggest an SFN clinical diagnosis for IENFD measurement.
• Patients who were over 18 years of age.
• Patients who were followed at Brest University Hospital.
• Patients who did not refuse to be included in the study.

Patients were classified in the SFN group when an IENFD lower than the 5th percentile (8) was measured in skin biopsies. The other patients (IENFD not in favor of SFN) were included in the control group. The measurement of the IENFD was performed by an only investigator (PM).

IENFD study was performed according to international recommendations (3, 4). Three-mm punch skin biopsies were taken at the distal leg (10 cm above the lateral malleolus). Additional biopsies were taken from the proximal thigh (20 cm below the anterior iliac spine). Specimens were immediately fixed in 4% paraformaldehyde for 24 h at 4°C, then kept in a cryoprotective solution over night, and serially cut with a cryostat. Each biopsy yielded about 50 vertical 50-μm sections. The first and last few sections were not used for nerve examination because of possible artifacts. Immunostainings were performed with an anti-PGP9.5 polyclonal antibody (Ultraclone, Isle of Wight, UK, dilution 1/800) then FITC goat anti-rabbit IgG (1/50) (Zymed, San Francisco, CA, USA) as secondary antibody.

The first step of the microscopic study was the measurement of the epidermal length in millimeter by computerized image software. Counting is realized at 400 magnification under immunofluorescence microscope. The quantification of nerve fibers was performed for each biopsy on 3 sections taking the mean. Normative reference values were those that were defined by the worldwide study (4).

The collected parameters were as follows from the patient file performed in our expert center according to our standardized 1-day check-up: age, sex, information from biopsy analysis (including IENFD at the distal and proximal leg), medical history, medications, and biological parameters at the moment of the biopsy (including blood cell counts, renal and liver function, autoantibodies, and viral serologies).

The protocol was accepted by the local ethics committee of Brest University Hospital (B2019CE.44) and registered on clinicaltrials.org (NCT04170205).

Descriptive statistics were presented as the means and standard deviations (SDs) for the quantitative variables and as percentages for the qualitative variables. For group comparisons (SFN vs. control), Chi2 or the Mann–Whitney test was applied as appropriate. A *p* < 0.05 was considered significant.

## Results

### Causes of SFN

Three hundred and fifty-one patients were included in this study. Most of these patients were followed in the dermatology department, but some of the patients were followed in the rheumatology, internal medicine, neurology, or pain management departments. Twenty-eight patients were excluded from the study because there were too numerous missing data in their medical records. Of the remaining 323 patients, 162 were diagnosed with SFN according to the IENFD and were included in the SFN group. The other 161 patients were included in the control group. The characteristics of the patients are presented in Table 1. The group of patients with SFN was made of 45.7% men and 54.3% women. There was no statistically significant difference in regard to sex distribution between the 2 groups. The patients in the SFN group (56 years ± 16) were younger than the patients in the control group (69 years ± 13) (*p* < 0.0001).

**TABLE 1:** Demographic and histological characteristics of the 323 included patients.

	Patients with SFN (*N* = 162)	Control patients (*N* = 161)	*P*
Men N (%)	74 (45.7)	57 (35.4)	0.06
Women N (%)	88 (54.3)	104 (64.6)	
Mean age years (SD)	56 (16)	69 (13)	<0.0001
IENF proximal mean/mm (SD)	4.8 (2.5)	5.7 (2.5)	0.001
IENF distal mean/mm (SD)	2.3 (1.6)	4.4 (1.9)	<0.0001

**FIGURE 1:**
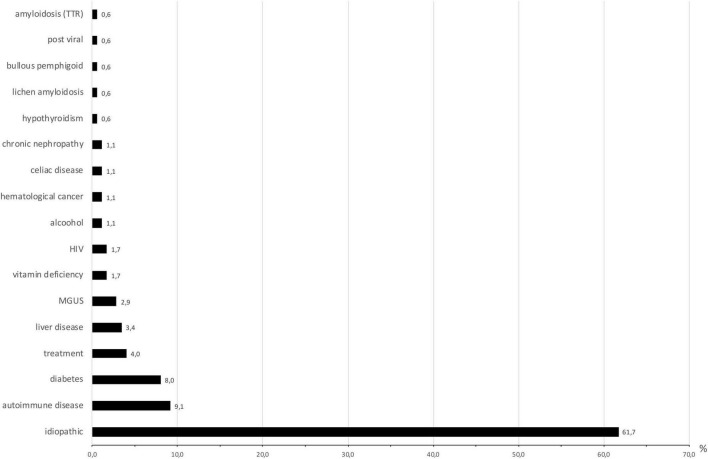
Causes of small-fiber neuropathy (expressed in percentage). *N* = 175 because some patients had more than one cause identified. MGUS, monoclonal gammopathy of undetermined significance; HIV, human immunodeficiency virus; TTR, transthyretin.

**FIGURE 2:**
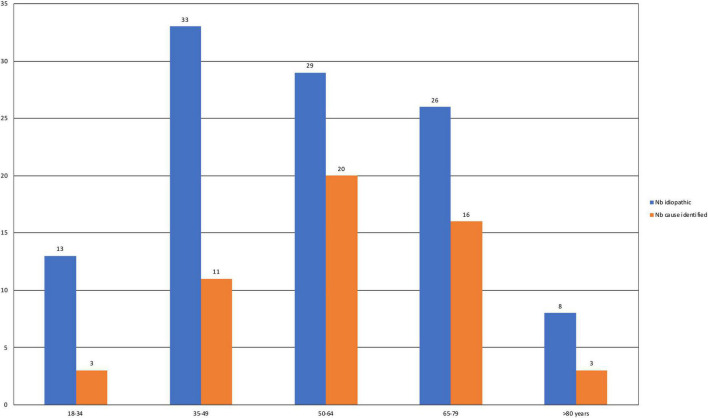
Numbers of patients by age group for those with idiopathic SFN and SFN with an identified cause. The chi2 test was used for comparisons between the two groups.

The identified causes of SFN are reported in Figure 1. Eight patients had 2 causes that could explain SFN, and two had 3. Consequently, several etiologies were reported for these ten patients. In total, the identified causes were 16 autoimmune diseases (9.4%), 14 diabetes or glucose intolerance (8%), 7 medications (4%), 6 liver diseases (3.4%), and 5 monoclonal gammopathies of undetermined significance (MGUS) (2.9%). Autoimmune diseases included Sjögren syndrome (6), lupus (1), sarcoidosis (1), cryoglobulinemia (2), rheumatoid arthritis (1), non-classified connective tissue disease (1), CREST syndrome (1), inflammatory bowel disease (1), and Sharp syndrome (2). Liver diseases were divided into viral hepatitis B (1) and C (1), hemochromatosis (1), iron overload (2) and cirrhosis (1). Medications reported as associated with SFN were chemotherapy protocols (2), adalimumab (1), risedronate (1), amlodipine (1), hormonal treatment (1) and tyrosine kinase inhibitors (1). No cause was identified for 108 patients (66.7%), and SFN was classified as idiopathic in these cases. This absence of identified cause appeared more frequent in younger populations, but it was not statistically significant (*p* = 0.31) (Figure 2).

### Skin biopsies

As reported in Table 1, the distal and proximal IENFD were significantly lower in the SFN group than in the controls, according to the selection criteria. Semiquantitative subepidermal nerve fiber density (SENFD) analysis was performed in all patients. The global analysis of all subepidermal densities found a statistically significant difference between the SFN and control groups for distal analysis (*p* = 0.02). No difference was found for proximal analyses (*p* = 0.45). Lymphoid infiltrates were investigated in all skin biopsies and were present in 38 patients with SFN and 30 control patients (*p* = 0.29). Amyloidosis and transthyretin amyloidosis were searched for in all samples, but amyloidosis was detected in only one biopsy without any specificity for transthyretin amyloidosis.

### Medical history and treatments

The medical histories of patients with SFN and controls are presented in Table 2. SFN patients less frequently had histories of stroke (1.2 vs. 8.1%; *p* = 0.004), hypertension (24.1 vs. 34.2%; *p* = 0.046), and dyslipidemia (8% vs. 17.4%; *p* = 0.012) than controls but more frequently had cirrhosis (2.5 vs. 0%; *p* = 0.045) and atopic dermatitis (6.2% vs. 1.2%; *p* = 0.019). Current or former smokers were more numerous in the SFN group (26.5%) than in the control group (16.1%) (*p* = 0.02).

**TABLE 2:** Medical history and treatments in the group of patients with SFN and the control group.

	Patients with SFN (*N* = 162) Number (%)	Control patients (*N* = 161) Number (%)	*P*
**Medical past N (%)**			
Stroke	2 (1.2)	13 (8.1)	**0.004**
Alcoholism	6 (3.7)	2 (1.2)	0.15
Cancer	24 (14.8)	27 (16.8)	0.63
Cardiopathy	14 (8.6)	21 (13)	0.20
Cirrhosis	4 (2.5)	0 (0)	**0.045**
Atopic dermatitis	10 (6.2)	2 (1.2)	**0.019**
Diabetes type 2	20 (12.3)	14 (8.7)	0.29
Dyslipidemia	13 (8.0)	28 (17.4)	**0.012**
Dysthyroidism	20 (12.3)	11 (6.8)	0.09
Fibromyalgia	3 (1.9)	5 (3.1)	0.47
Hypertension	39 (24.1)	55 (34.2)	**0.046**
Autoimmune diseases	42 (25.9)	47 (29.2)	0.51
Psychiatric disorders	24 (14.8)	26 (16.1)	0.74
Tobacco	43 (26.5)	26 (16.1)	**0.023**
**Medication N (%)**			
Anti-diabetic medications	13 (8.0)	10 (6.2)	0.53
Central antihypertensive drugs	2 (1.2)	2 (1.2)	1.00
Antiplatelet drugs	21 (13.0)	27 (16.8)	0.34
Antiarrhythmics	2 (1.2)	5 (3.1)	0.25
Angiotensin II receptor blockers	19 (11.7)	28 (17.4)	0.15
Vitamin K antagonists	9 (5.6)	7 (4.3)	0.62
Benzodiazepines	34 (21.0)	48 (29.8)	0.07
Beta blockers	14 (8.6)	16 (9.9)	0.69
Diuretics	23 (14.2)	16 (9.9)	0.24
Thyroid hormone	20 (12.3)	12 (7.5)	0.14
Anti uricemic agents	6 (3.7)	1 (0.6)	0.06
Lipid lowering agents	19 (11.7)	38 (23.6)	**0.005**
Angiotensin converting enzyme inhibitors	10 (6.2)	14 (8.7)	0.39
Calcium channel blockers	15 (9.3)	16 (9.9)	0.84
Insulin	6 (3.7)	3 (1.9)	0.32
Proton pump inhibitors	30 (18.5)	34 (21.1)	0.56
Direct Xa inhibitors	7 (4.3)	3 (1.9)	0.20
Antipsychotics	3 (1.9)	6 (3.7)	0.31
Opioids	20 (12.3)	26 (16.1)	0.33
Paracetamol	24 (14.8)	40 (24.8)	**0.024**

The chi2 test was used for comparisons between the SFN and control groups. *p* <0.05.

The mean number of drugs taken by patients was 3.4 ± 3.1 in the SFN group and 4.1 ± 3.4 in the control group (*p* = 0.08). Lipid-lowering drugs (statins and fibrates) were more frequently taken by patients in the control group (23.6%) than in the SFN group (11.7%) (*p* = 0.01), and paracetamol was more frequently taken by 24.8% of the control group and 14.8% of the SFN group (*p* = 0.02) (Table 2).

Among the biological parameters, the hemoglobin concentration was higher in the SFN group (14.0 g/dl ± 1.9) than in the control group (13.0 g/dl ± 1.4) (*p* = 0.01). Lymphocyte counts were also higher in the SFN group than in the control group (SFN group: 2.0 giga/l ± 1.0; control group: 1.8 giga/l ± 0.74) (*p* = 0.03). Among iron parameters, serum iron concentration (SFN group: 14 μmol/l ± 6.1; control group: 16 μmol/l ± 6.1) (*p* = 0.03) and transferrin saturation (SFN group: 25% ± 14; control group: 32% ± 13) (*p* = 0.02) were lower in the SFN group. Among vitaminic parameters, vit D levels were lower in patients with SFN (38 ± 20) than in controls (60 ± 31) (*p* < 0.04) (Table 3).

**TABLE 3:** Biological parameters recorded at the time of skin biopsy as part of the explorations of conditions associated with possible SFN.

Biological parameters mean ± SD	Patients with SFN (*N* = 162)	Control patients (*N* = 161)	*P*
Hemoglobin (g/dl)	14 ± 1.9	13 ± 1.4	**0.01**
Platelets (giga/l)	235 ± 70	246 ± 75	0.15
Leukocytes (giga/l)	6.8 ± 2.2	6.4 ± 2	0.14
Lymphocytes (giga/l)	2 ± 1	1.8 ± 0.74	**0.03**
Glucose (mmol/l)	5.6 ± 2.1	5.4 ± 2.7	0.31
Angiotensin converting enzyme (U/l)	30 ± 17	32 ± 17	0.65
Serum iron (μmol/l)	14 ± 6.1	16 ± 6.1	**0.03**
Transferrin (μmol/l)	28 ± 7.3	26 ± 4.4	0.37
Transferrin saturation (%)	25 ± 14	32 ± 13	**0.02**
Ferritin (ug/l)	178 ± 354	189 ± 240	0.10
Vitamin B6 (pmol/l)	17 ± 15	48 ± 147	0.24
Vitamin B12 (nmol/l)	280 ± 106	346 ± 327	0.38
Vitamin D (nmol/l)	38 ± 20	60 ± 31	**0.04**
TSH (mUI/l)	1.9 ± 1.2	2 ± 1.2	0.78
Total serum proteins (g/L)	68 ± 8.4	68 ± 8.6	0.92
Anti-tissue transglutaminase (UA)	0.74 ± 5	0.62 ± 4	0.70

The chi2 test was used for comparisons between the SFN and control groups. *p* <0.05.

HIV serology was positive in 3 patients with SFN (1.8%) vs. 1 in the control group (0.6%) (*p* = 0.41). HCV serology was positive in 3 patients from the SFN group (1.9%) and none in the control group (*p* = 0.27). HBV serology was positive for 20 patients in the SFN group (12.5%) and 10 in the control group (6.2%) (*p* = 0.45).

Among immunological explorations, antinuclear antibodies were present in 71 patients in the SFN group (43.8%), with a titer greater than 1/320 in 24 patients. In the control group, they were found in 57 patients (35.4%), with a titer higher than 1/320 for 25 patients. There was no difference between the two groups regarding the presence of antinuclear antibodies (*p* = 0.29).

Fabry disease was investigated in 75 patients in the SFN group and 53 in the control group, and no cases were identified.

Chest X-ray was performed on 101 patients in the SFN group and never revealed specific abnormalities. In the control group, 94 chest X-rays were performed, and 1 case of sarcoidosis was detected.

## Discussion

In the present study, 61.2% of SFNs remained idiopathic, which is close to the mean previously reported in other publications (approximately 50%) (6, 9, 10), which ranged from 22.6 to 73% (11, 12). In our study, we found numerous putative causes of SFN that were previously reported, such as diabetes, MGUS, Sjogren disease, and other autoimmune diseases (6, 9, 10, 13, 14), but we identified unknown putative causes.

There are only a few studies evaluating the prevalence of SFN-associated causes. Rather than studies on large cohorts (6, 10, 15, 16), studies on SFN cohorts were more frequently focused on well-defined populations [idiopathic (17), alcoholic (11, 18), Sjogren syndrome (19, 20), diabetes (21), cryoglobulinemia (22), and Fabry disease (23)]. Some etiologies, such as vaccinations (24) or medications (5, 7), were described only in case reports or case series. In our study, 7 medications were reported to have a possible relationship with an SFN, among which 4 are not yet known to be associated with SFN (risedronate, amlodipine, progestogen, and imatinib). The chronology was compatible with a delay in the appearance of clinical symptoms between 1 and 6 months after the introduction of the treatment. In one case (imatinib, a tyrosine kinase inhibitor), the underlying hematologic disease (chronic myelogenous leukemia) was also a possible cause of SFN. These results need to be confirmed by future studies.

In our hands, the comparison between the SFN and control groups highlights the possible role of tobacco consumption in SFN pathogenesis since there was a significantly higher number of tobacco users in the SFN group (26.5%) than in the control group (16.1%). To our knowledge, a history of smoking has never been studied in SFN patients. The effects of tobacco could be directly related to one or several compounds of tobacco, and an indirect effect could be related to the aggravating role of tobacco in diabetic neuropathy (25), chemotherapy-induced peripheral neuropathy (26), or HIV neuropathy (27). Based on these results, we might advise patients with SFN to stop smoking.

The lower cardiovascular comorbidity (hypertension, dyslipidemia, and stroke) observed in the SFN group is probably secondary to the younger age in this group. To date, a negative impact of vascular disease on neuropathy has been acknowledged (25). A lower use of lipid-lowering drugs (statins and fibrates) and paracetamol (28) in the SFN group could be a consequence of these age differences between the two groups. Statins were previously related to an increase in the frequency of SFN and polyneuropathies (29), while a specific study on the SFN population was in favor of an absence of relation (30). Angiotensin II receptor blockers have been shown to have a neuroprotective effect (31, 32). This protective effect was not found in our study, probably due to the small number of patients. Antiplatelet drugs have also been reported to have possible neuroprotective effects (33).

The analysis of biological parameters showed significant differences in hemoglobin, lymphocytes, iron parameters, and vitamin D. It is likely that hematological differences are related to the age difference between the two groups. Regarding the iron parameters, both iron deficiency and overload were found to be more frequent in the SFN group.

More interestingly, lower blood concentrations of vitamin D were observed in the SFN group, which could justify a systematic screening of patients suspected of having SFN and treatment in case of a proven deficiency. Further studies are needed. The role of the interplay between vitamin D and its receptor (VDR) and known specific pain signaling pathways, as well as their role in pruritus and pain sensitizations (34), supports the role of vitamin D deficiency in SFN, while vitamin D receptors are present on neurons, oligodendrocytes and glial cells (35).

The SFN pathophysiology is poorly known. Small-fiber degeneration and nociceptor sensitization are the main observed mechanisms (36). Vitamin D insufficiency or disorders of iron metabolism may have a negative impact on both innate and adaptive immunity and the development and function of the nervous system (35, 37–39). Long-term exposure to tobacco increases pain sensitivity due to nicotinic acetylcholine receptor desensitization and neuronal plastic changes (40).

The main limitation of this study is its retrospective nature. The monocentric design is another limitation. However, our population did not seem to have specific characteristics that were observed in some other studies reported, including an excessively elevated number of specific etiologies, such as sarcoidosis (9). Another point is that patients usually underwent only one check-up, while the repetition of certain examinations (such as the search for diabetes) seems valuable in the screening of causes associated with idiopathic neuropathies (17, 41). The absence of systematic electrophysiological explorations or quantitative sensory testing (QST) could be considered a limitation, but a diagnosis of SFN based on the IENFD measurement is currently considered sufficient. Finally, we cannot exclude that some patients in the control group may have an SFN with current normal IENFD (42) because it is represented by patients with signs and symptoms of small-fiber neuropathy having an intraepidermal nerve fiber density higher than the 5th percentile.

It remains interesting to find new etiologies of SFN. Recently, post-COVID or post-Lyme treatment SFN were reported (43–45).

In conclusion, we performed a study on patients with SFN to search for possible causes of SFN. Smoking was shown to be associated with SFN, so we should advise patients to stop smoking. Further work exploring this association appears to be necessary to determine whether it is a cause of SFN or an aggravating factor. Vitamin D deficiency and the use of some drugs could be considered putative causes, but there is a need for further studies.

## Data availability statement

The raw data supporting the conclusions of this article will be made available by the authors, without undue reservation.

## Ethics statement

This study involving human participants was reviewed and approved by Local Ethics Committee of Brest University Hospital (B2019CE.44). The patients/participants provided their written informed consent to participate in this study.

## Author contributions

MF performed the study and wrote the manuscript. LM was supervisor of the study. SG and LM provided the clinical data. PM provided the histological data. EB and A-SF performed the statistical analysis. All authors contributed to the article and approved the submitted version.
